# Construction Notes

**Published:** 1895-03-30

**Authors:** 


### CONSTRUCTION NOTES.

### New Hospital at Berkeley.

The commodious house and premises recently purchased by Lord Fitzhardinge for the Berkeley Hospital, Gloucestershire, situate in Maryport Street, have been thoroughly repaired, renovated, and altered to suit the requirements of a hospital. On the ground floor there is a spacious entrance hall, committee room, matron's room, day room for patients, 19 ft. by 16 ft., large kitchen and offices, and a mortuary has been erected at the back. On the first floor is a roomy landing, men's ward, 22 ft. by 16 ft., women's ward, special ward for accidents, matron's bed-room, bath-room, fitted with hot and cold water. The rooms are lofty and fitted with an excellent arrangement of gas fittings. The second floor contains bed-rooms for the officials and servants. There is a pleasure garden and kitchen garden at the rear, the drains are all fitted with patent traps, and the sanitary arrangements are complete in every respect. The work has been carried out by Mr. F. W. Ford, of Uley, under the superintendence of Mr. R. Ransford, Lord Fitzhardinge's surveyor, at the cost of about £300.

